# An Assessment of Sikh Turban’s Head Protection in Bicycle Incident Scenarios

**DOI:** 10.1007/s10439-023-03431-7

**Published:** 2024-02-02

**Authors:** Xiancheng Yu, Gurpreet Singh, Amritvir Kaur, Mazdak Ghajari

**Affiliations:** 1 HEAD Lab, Dyson School of Design Engineering, Imperial College London, London, UK; 2 Department of Mechanical Engineering, University of Sheffield, Sheffield, UK; 3 Department of Materials, Imperial College London, London, UK; 4 Sikh Scientists Network, London, UK; 5 Dr Kaur Projects Ltd, London, UK

**Keywords:** Traumatic brain injury, Sikh turbans, Oblique impact, Rotational acceleration, Translational acceleration

## Abstract

Due to religious tenets, Sikh population wear turbans and are exempted from wearing helmets in several countries. However, the extent of protection provided by turbans against head injuries during head impacts remains untested. One aim of this study was to provide the first-series data of turbans’ protective performance under impact conditions that are representative of real-world bicycle incidents and compare it with the performance of bicycle helmets. Another aim was to suggest potential ways for improving turban’s protective performance. We tested five different turbans, distinguished by two wrapping styles and two fabric materials with a size variation in one of the styles. A Hybrid III headform fitted with the turban was dropped onto a 45 degrees anvil at 6.3 m/s and head accelerations were measured. We found large difference in the performance of different turbans, with up to 59% difference in peak translational acceleration, 85% in peak rotational acceleration, and 45% in peak rotational velocity between the best and worst performing turbans. For the same turban, impact on the left and right sides of the head produced very different head kinematics, showing the effects of turban layering. Compared to unprotected head impacts, turbans considerably reduce head injury metrics. However, turbans produced higher values of peak linear and rotational accelerations in front and left impacts than bicycle helmets, except from one turban which produced lower peak head kinematics values in left impacts. In addition, turbans produced peak rotational velocities comparable with bicycle helmets, except from one turban which produced higher values. The impact locations tested here were covered with thick layers of turbans and they were impacted against flat anvils. Turbans may not provide much protection if impacts occur at regions covered with limited amount of fabric or if the impact is against non-flat anvils, which remain untested. Our analysis shows that turbans can be easily compressed and bottom out creating spikes in the headform’s translational acceleration. In addition, the high friction between the turban and anvil surface leads to higher tangential force generating more rotational motion. Hence, in addition to improving the coverage of the head, particularly in the crown and rear locations, we propose two directions for turban improvement: (i) adding deformable materials within the turban layers to increase the impact duration and reduce the risk of bottoming out; (ii) reducing the friction between turban layers to reduce the transmission of rotational motion to the head. Overall, the study assessed Turbans’ protection in cyclist head collisions, with a vision that the results of this study can guide further necessary improvements for advanced head protection for the Sikh community.

## Introduction

Turbans are an integral part of Sikh religion’s identity that cannot be compromised or replaced with hats or helmets according to religious tenets. A Sikh takes about 15 to 30 min everyday to wear a turban over his/her head, by systematically wrapping it around the forehead and largely covering the skull and parts of the neck and ears. World War I and II are the best examples of Sikhs’ credibility to carry out dangerous combat missions, without replacing their turbans with helmets. This has enabled the Sikhs to gain exemptions in various countries to carry-on wearing turbans instead of helmets. These include the United Kingdom (Religious exemption act), India (Sikhs exempt as per Section 129 of the Central Motor Vehicles Act), Canada (Alberta, British Columbia, Manitoba, and Ontario state), Denmark, New Zealand, Sweden, and Thailand.

Despite the laws facilitating Sikhs to enjoy the religious freedom of wearing turbans for both bicycle and motorcycle journeys, there has been no efforts to understand the safety of Sikh riders with turbans. This lack of understanding has prevented any efforts to improve the potential head protection of turbans and to extend this to other exposed areas of the head.

The aim of this study is to conduct the first assessment of the Sikh turbans for their protection against head injuries in bicycle incident scenarios. In this initial study, we perform impact tests on five different turbans with varying styles and materials using a method that represents bicycle impact conditions [1]. We test whether the performance of turbans in reducing injury metrics based on head kinematics, such as peak linear and rotational acceleration of the head, is comparable with conventional bicycle helmets. In addition, we further analyze the test results to understand the potential mechanisms by which turbans can provide head protection during impacts. This analysis can help determine ways for improving the head protection capability of turbans.

## Methods

### The Sikh Turbans

**Fig. 1:**
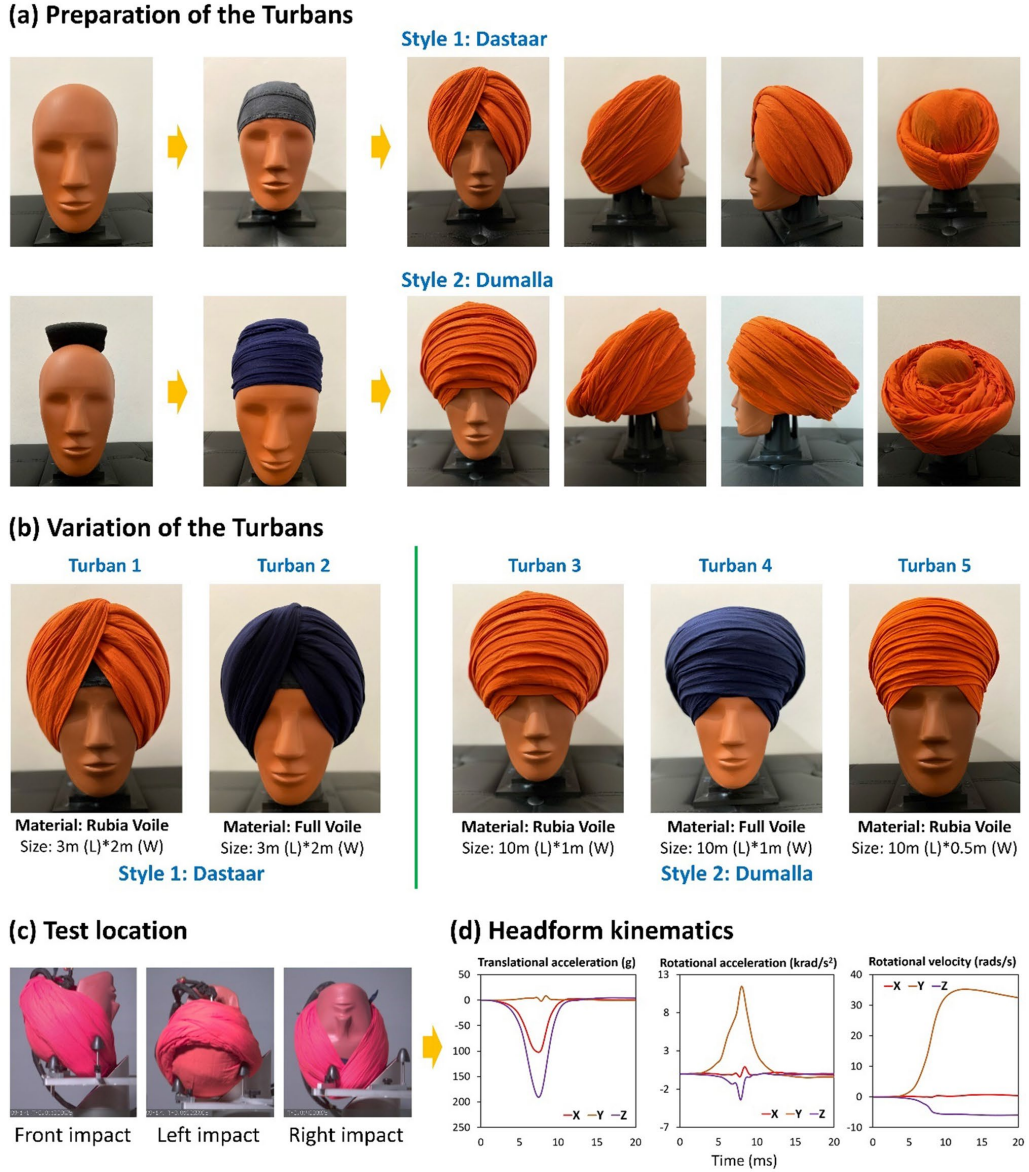
**a** Preparations of the turbans using two different styles. **b** The five turbans used here. **c** Turbans-tied headform were impacted onto the oblique anvil at three locations. **d** For each test, three translational accelerations, three rotational accelerations, and three rotational velocities time-history data were recorded

We used two different styles to wrap the turban allowing us to test the most common types of turbans worn by Sikhs, shown in Fig. 1. Style 1 is called Dastaar or Pagadi. It is the most common modern turban style that is comfortably recognized and denoted throughout the world among the Sikh male population. This is the most common and constantly improvised version of the turban style, with different variations in length and material of cloth. Style 2 is called Dumalla, which is referred to the traditional warrior turban. Dumalla is a niche and complicated turban style, shared by both male and female Sikhs. This style had historically been referenced to baptized Sikh soldiers (Khalsa) who were warriors and supposedly denounced the use of any other head protections such as helmets. Dumalla consists of two turbans, a smaller base cloth (about 3 m in length and 0.5 m in width) and a longer outer cloth (usually 10 meters or more long and at least 0.5 meters in width) that is placed in layers around the head multiple times in a pseudo-circular fashion forming a cylindrical shape, starting from the bottom of forehead and progressing vertically above. Dumalla’s pattern of layers may or may not be clear, but it is recognized, irrespective of any variations in the style, by its large size and unique style.

For both styles, we used two different turban fabric materials, including the Rubia Voile (orange color in Fig. 1) and Full Voile (dark navy-blue color). The Rubia Voile material is mainly composed of pure cotton, often mixed with linen and polyester in varying amounts, depending on quality and source of production. It is a modern fabric adopted at large by Sikhs for tying turbans that look thick and voluminous, while still being lighter than pure Rubia fabric. The Full Voile material is composed of pure cotton, sometimes mixed with linen in varying amounts. It is lighter in weight and softer than Rubia Voile and is often chosen by Sikhs for tying compact and light-weighted turbans.

In total, we tested 5 different turbans, as shown in Fig. 1b and Table 1. Turbans 1 and 2 were wrapped with style 1 (Dastaar or Pagadi) using Rubia Voile and Full Voile, respectively. The size of both fabric materials was 3 m in length and 2 m in width, representing the average fabric size of style 1. After wrapping, this turban had 5 layers on the left side forming a clear layered pattern (Fig. 1b). Turbans 3 and 4 were wrapped with style 2 (Dumalla) using both fabric materials with a 10 m length and 1 m width. In addition, to represent the female population, we made turban 5 with style 2 using a smaller Rubia Voile fabric: 10 m in length and 0.5 m in width. Turbans 3, 4, and 5 have a pseudo-circular fashion with multiple layers (Fig. 1b).

**Table 1:** Summary of the turbans and impact location

Type	Style	Material	Fabric dimension (length*width)	Impact location
Turban 1	Dastaar Dastaar	Rubia Voile	3 m × 2 m	Front, left, right
Turban 2		Full Voile	3 m × 2 m	Front, left
Turban 3	Dumalla	Rubia Voile	10 m × 1 m	Front, left
Turban 4	Dumalla	Full Voile	10 m × 1 m	Front, left
Turban 5	Dumalla	Rubia Voile	10 m × 0.5 m	Front, left

### Oblique Impact Tests Representing Cyclist Head Collisions

We tested turbans under oblique impacts at three different impact locations (Fig. 1c), representing a wide range of head impacts occurring in real-world cycle incidents [2]. A recent review of studies on cycle incidents found that the angle between the head velocity and the normal to the impact surface is concentrated around 30° to 50° [2]. In addition, this study found that the head impact speed (i.e., the magnitude of the head velocity) is concentrated around 5 to 8 m/s. Finally, it found that the side and front regions of the head are the most frequently impacted. This review supports the impact conditions used in recent studies to assess the performance of a range of bicycle helmets [1, 3, 4]. These conditions included impacts to the side and front of the helmets through dropping a helmeted HIII headform at a 6.3 m/s (~23 km/h) impact speed onto an anvil with a 45° inclined surface covered with a grade 40 abrasive paper. We used these conditions to test the turbans, allowing us to compare the protective performance of the turbans to bicycle helmets.

The turbans were wrapped onto a Hybrid III (HIII) 50th percentile male dummy headform by a Sikh male with several years of experience in wrapping different turban styles. A nine-accelerometer package (NAP) was installed within the headform to measure the translational and rotational accelerations of the headform during the impact. The accelerometers were mounted in a 3-2-2-2 array [5]. The accelerometers were sampled by a datalogger at 50 kHz frequency. We filtered the acceleration data, using a fourth-order Butterworth filter at a cut-off frequency of 1 kHz, as suggested in [6–8]. We calculated the rotational velocity by integrating the components of the rotational acceleration in the head-fixed coordinate system vs. time [5, 9].

A high-speed video camera was used to record the impacts at 1770 frames per second. After each test, we checked the high-speed video to make sure that each test was performed as intended and the headform was not displaced on the platform during the free fall.

We tested the turbans at three impact locations: front, left side, and right side (Fig. 1c). The turban style 1 has a different type of layering at the left and right sides. During the tests, we found that the right side produced much higher accelerations than the left side, producing readings close to the limit of the accelerometers. Therefore, to avoid accelerometer damage, we only tested turban 1 at the right side. To keep the data synchronous, all turbans were tested at the front and left sides, as detailed in Table 1. Each test was repeated three times with a newly wrapped turban. There was no apparent damage to the turbans after the impacts, aside from a few minor friction marks. Consequently, due to the limited supply of turban fabric, we decided to reuse some of the fabric samples, but we re-tied the fabric after each impact to ensure that the same area of the fabric is not subjected to more than one impact.

### Brain Injury Metrics Based on Head Kinematics

The kinematics of the headform, measured with the head-mounted sensors, was processed to obtain three metrics that are often used to predict the risk of different types of brain injuries: peak translational acceleration (PTA), peak rotational acceleration (PRA), and peak rotational velocity (PRV). PTA is proportional to the peak force applied to the head and is suitable for predicting the risk of skull fractures and focal brain injuries [10, 11]. PTA is used in all helmet standards, e.g., the European cycle helmet standard EN1078 which defines a 250 g limit for it. Direct or indirect forces can cause rotation of the head, leading to large brain deformations and stretching of different structures such as vessels. In order to extend the assessment of the turban protection against injuries caused by head rotation, we used PRA and PRV. PRA has been suggested as a metric for predicting subdural haematoma (SDH) [12, 13]. PRV has been adopted to predict the risk of diffuse axonal injuries [14, 15]. Since these pathologies are reported for cyclists who sustain a TBI [2, 16], we used all three metrics to evaluate the performance of the turbans and compare it with conventional bicycle helmets.

To investigate the effects of the turban and impact location on turban’s protective performance, we performed two-way ANOVA using turban type and impact location as the factors and the injury metrics as the outcome measure.

## Results

### Head Kinematics and Injury Metrics

**Fig. 2:**
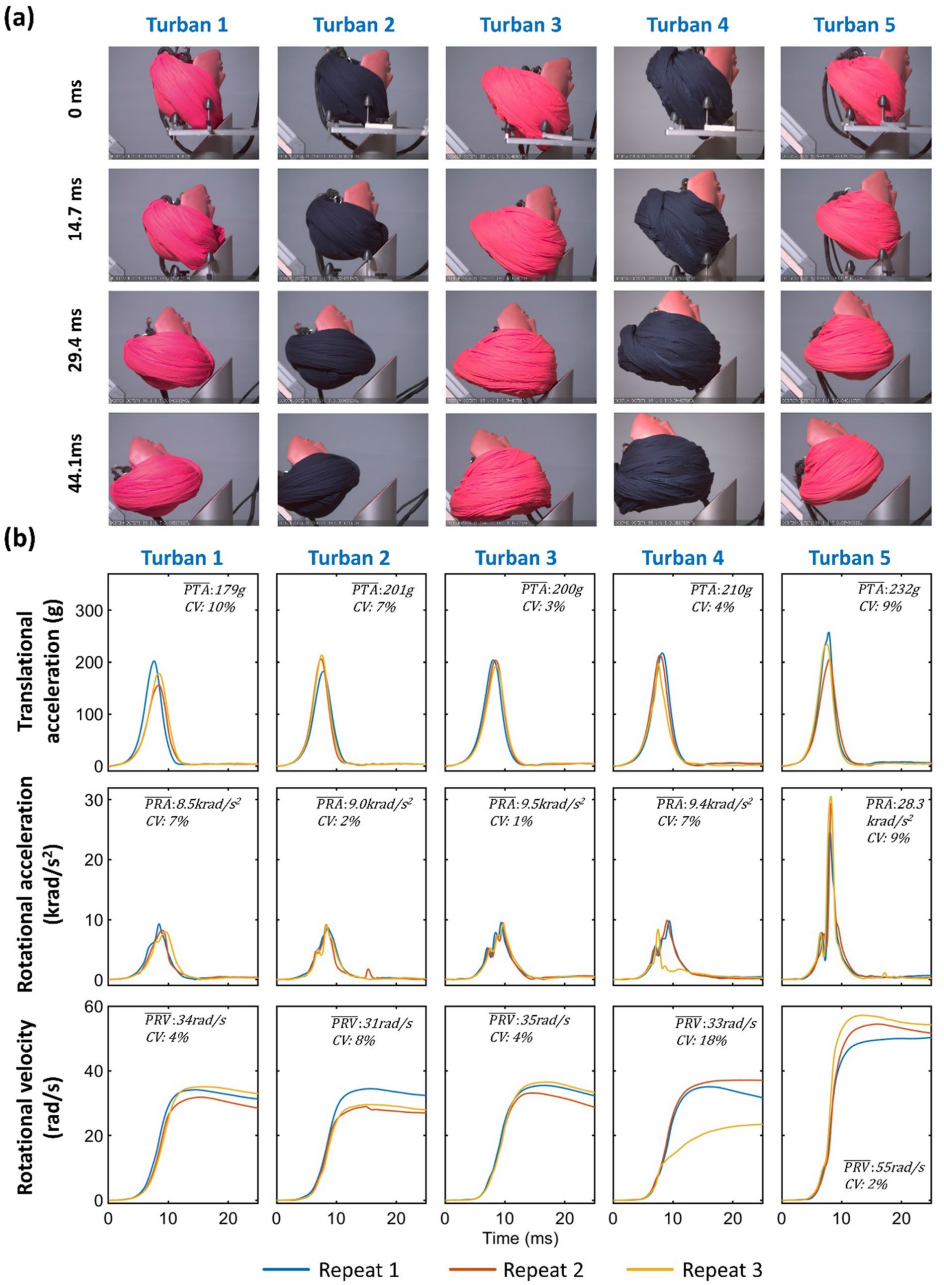
Front impact results: **a** Snapshots from the high-speed videos of all turbans under front impact. **b** The resultant translational acceleration, rotational acceleration, and rotational velocity time-histories of three repeats for all turbans under front impact. The mean value and CV of the three repeats for each injury metric are shown in each subplot

**Fig. 3:**
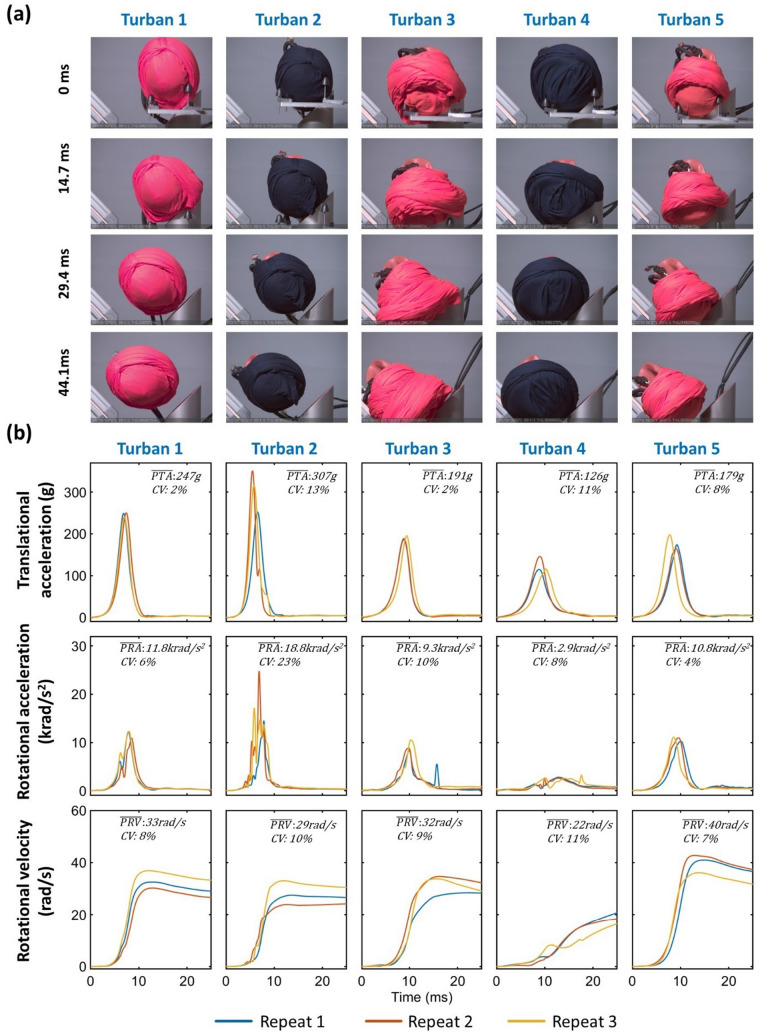
Left impact results: **a** Snapshots from the high-speed videos of all turbans under left-side impact. **b** The resultant translational acceleration, rotational acceleration, and rotational velocity time-histories of three repeats for all turbans under left-side impact. The mean value and CV of the three repeats for each injury metric are shown in each subplot

**Fig. 4:**
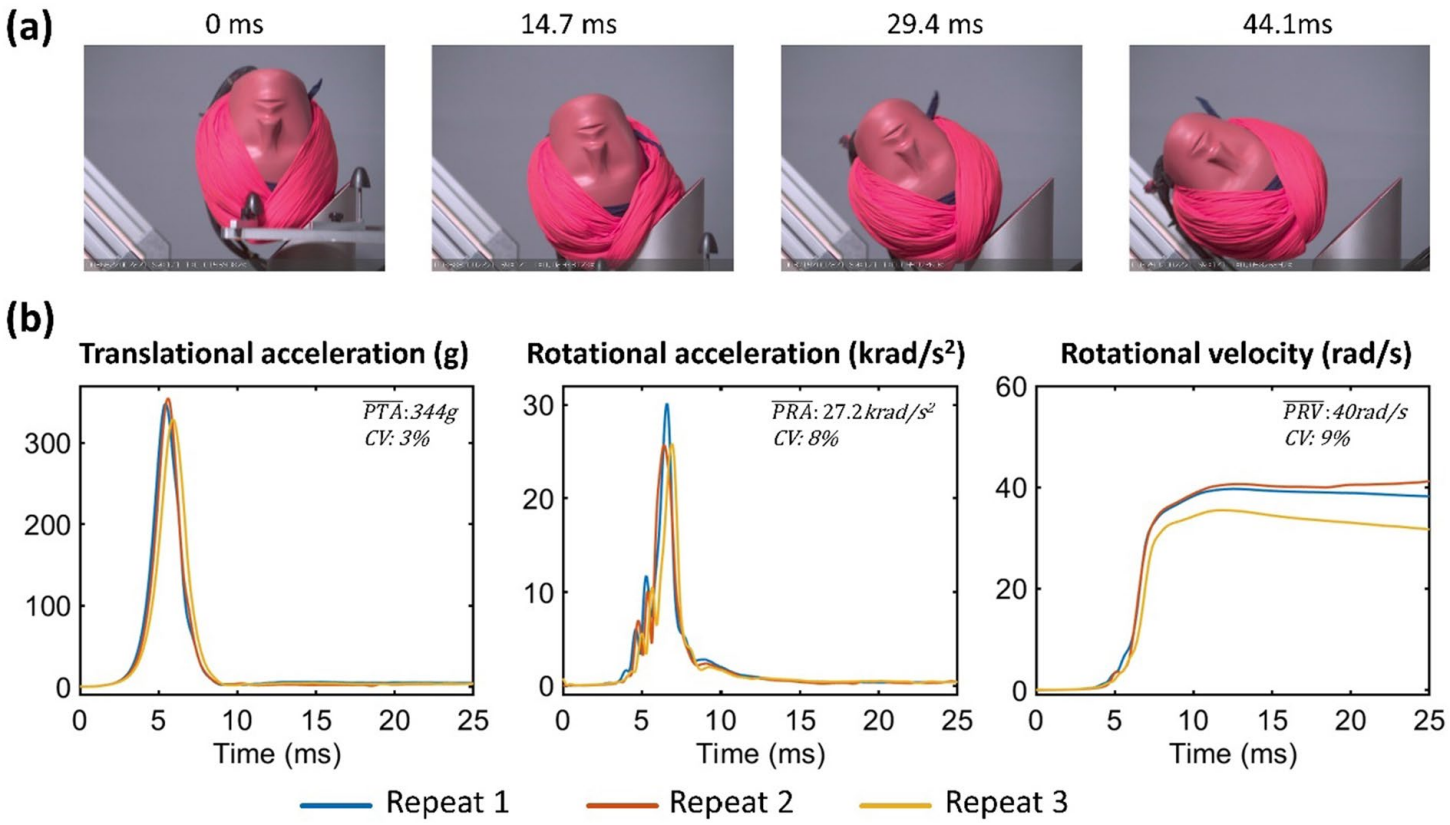
Right impact results: **a** Snapshots from the high-speed videos of turban 1 under right-side impact. **b** The resultant translational acceleration, rotational acceleration, and rotational velocity time-histories of three repeats for all turbans under right-side impact. The mean value and CV of the three repeats for each injury metric are shown in each subplot

For all impact locations and turbans, an inspection of the high-speed video confirmed that the impacts occurred at the intended location (Figs. 2, 3, and 4). During impact, the turban was compressed continuously, and following head rotation, it detached from the anvil. In all experiments, although slightly distorted, a freshly tied turban remained on the headform after the impact.

The time-histories of the resultant translational and rotational acceleration for all turbans and impact locations indicate the good repeatability of the majority of the impacts, with the coefficient of variation (CV) of peak values across the three repeats remaining below 10% (Figures 2, 3, and 4). However, the CV of PRV for turban 4 under front impact was 18%, and the CV for PRA for turban 2 under left impact was 23%, which indicate poor repeatability of these tests.

### The Turban Type Affects Injury Metrics, but this Depends on Impact Location

A two-way ANOVA showed that both the turban [F (4) = 22.46, *p* < 0.001] and the impact location [F (2) = 38.70, *p* < 0.001] have a significant effect on PTA. In addition, there is an interaction between the two factors[F (4, 22) = 21.43, *p* < 0.001] on PTA. In front impacts, the mean PTA was 204 g. Turban 5 produced significantly higher PTA (232 g) than other turbans while turban 1 produced the lowest PTA (179 g), showing 23% difference in mean PTA comparing worst and best performing turban. In left impact, the mean PTA (210 g) was similar to the front impact, while the range was much larger (126 g-307 g). Turban 2 produced very high PTA (307 g), while the PTA of turban 4 was only 126 g, showing a 59% difference in mean PTA. In right impact, we only tested turban 1, whose PTA (344 g) is the highest among all turbans and impact locations.

For PRA, the two-way ANOVA results showed that both the turban [F (4) = 39.45, *p* < 0.001] and the impact location [F (2) = 70.45, *p* < 0.001] have a significant effect, as well as their interaction [F (4, 22) = 36.79, *p* < 0.001]. In front impacts, the mean PRA was 12.9 krad/s^2^. Turban 5 produced much higher PRA (28.3 krad/s^2^) than other turbans while turban 1 produced the lowest PRA (8.5 krad/s^2^), indicating a 70% difference. In left impact, the mean PRA was 10.7 krad/s^2^. The range was very large (2.9-18.8 krad/s^2^). Turban 4 produced surprisingly low PRA (2.9 krad/s^2^), while the highest PRA was 18.8 krad/s^2^ for turban 2, showing an 85% difference. In right impact, we only tested turban 1, whose PRA (27.2 krad/s^2^) was the second highest among all turbans and impact locations.

For PRV, the two-way ANOVA results showed that both the turban [F (4) = 26.85, *p* < 0.001] and the impact location [F (2) = 13.75, *p* < 0.001] have a significant effect, as well as their interaction [F (4, 22) = 4.57, *p* < 0.01]. In front impacts, the mean PRV was 37.6 rad/s. Similar with PTA and PRA, turban 5 also produced the highest PRV (55 rad/s). The other turbans produced similar PRVs (31-35 rad/s), showing a 44% difference between the highest and lowest values. In left impact, the mean PRV was 31.2 rad/s. Again, turban 5 produced the highest PRV (40 rad/s). The PRV of turban 4 was 22 rad/s, which was the lowest among all turbans and impact locations, showing a 45% reduction compared with the worst performing turban. In right impact, we only tested turban 1, and its PRV was 40 rad/s.

## Discussion

We assessed the protective performance of Sikh turbans under impact conditions representing typical cyclist head collisions. We found that the style and fabric of the turban can have a considerable effect on the head injury metrics, PTA, PRA, and PRV. In addition, we found that for the same turban, impact on the left or right side of the head can produce very different head kinematics, showing the effects of turban layering in mitigating head injuries. This study provides the first assessment of Sikh turbans under oblique impacts, thereby providing insights into the protective performance of turban.

### Turbans’ Protective Performance

We found that the turban type influences PTA. In front impacts, turban 1 produced the lowest PTA and turban 5 the highest PTA, with a 23% reduction in PTA from turban 5 to 1. In left impacts, turban 4 produced the lowest PTA and turban 2 the highest PTA, with a 59% reduction in PTA. In front and left impacts, all turbans, except turban 2 in left impact, produced PTAs lower than the pass/fail limit prescribed in bicycle helmet standards (250 g) [17]. PTA is a predictor of skull fracture, and a PTA of 250 g is equivalent to a 40% risk of skull fracture [18]. Therefore, our results show that some turban types can greatly reduce this risk (e.g., turban 4 in left impact), but this is highly dependent on the impact location as we have seen for instance with turban 1, showing a 28% increase in PTA from left to right impact.

We also found that different turbans lead to a wide range of rotational head kinematics metrics, PRA and PRV. The turban type led to a 70% reduction in PRA in front impact (turban 2 vs 5) and 85% reduction in left impact (turban 4 vs 1). PRA is suggested as a predictor of subdural hematoma (SDH) with a 10 krad/s^2^ threshold determined from PMHS experiments [12, 13]. In front impacts, PRAs were close to this value but only turban 5 produced a PRA that exceeded this threshold. In side impacts, turbans 1, 2, and 5 produced PRAs larger than this threshold. In addition, PRV is suggested as a predictor of the risk of diffuse axonal injury (DAI), with a 46.5 rad/s as threshold for moderate-to-severe DAI [15]. All tests produced PRVs lower than this value, except for turban 5 under front impact.

The unhelmeted (bare head) impact test data under the same impact conditions are currently unavailable. In addition, such tests were not conducted due to the apparent concern of damaging the headform and the sensors. Therefore, to understand the performance of turbans compared with an unprotected head, we used data from a previous experimental work conducted by Cripton et al. [19]. They performed impacts on the Hybrid III headform at the forehead location by dropping it on a flat horizontal anvil. Among the impact scenarios, one involved a speed of 4.4 m/s, which is very close to the 4.5 m/s component of our impact velocity normal to the anvil. Since the translational acceleration of the headform is primarily determined by the normal impact speed [20], we compared the PTA from our tests with the test on the bare headform reported in Cripton et al.’s study. In their 4.4 m/s impact, the PTA was 471 g for the bare headform [19]. In contrast, turbans yielded PTAs ranging from 179 to 232 g in front impacts, representing a substantial reduction compared to bare head impacts. We should note the test has set up differences between Cripton et al.’ study and current study. Specifically, Cripton et al. used guided falls using a ball-arm attached to a mono-rail tower, while we used free falls, providing more freedom for the headform motion and therefore producing lower PTAs. A recent work studied the differences between guided fall and free fall to evaluate the response of headforms fitted with motorcycle helmets under 7.5 m/s impacts against a flat horizontal anvil [21]. They found that PTAs produced by free falls are 3 to 17% lower than guided falls. This difference is substantially less than the reduction in PTA produced by turbans (51 to 62% lower). Therefore, despite the differences between guided and free falls, the substantial difference in PTA indicates that turbans considerably reduce PTA compared to bare head impacts.

**Fig. 5:**
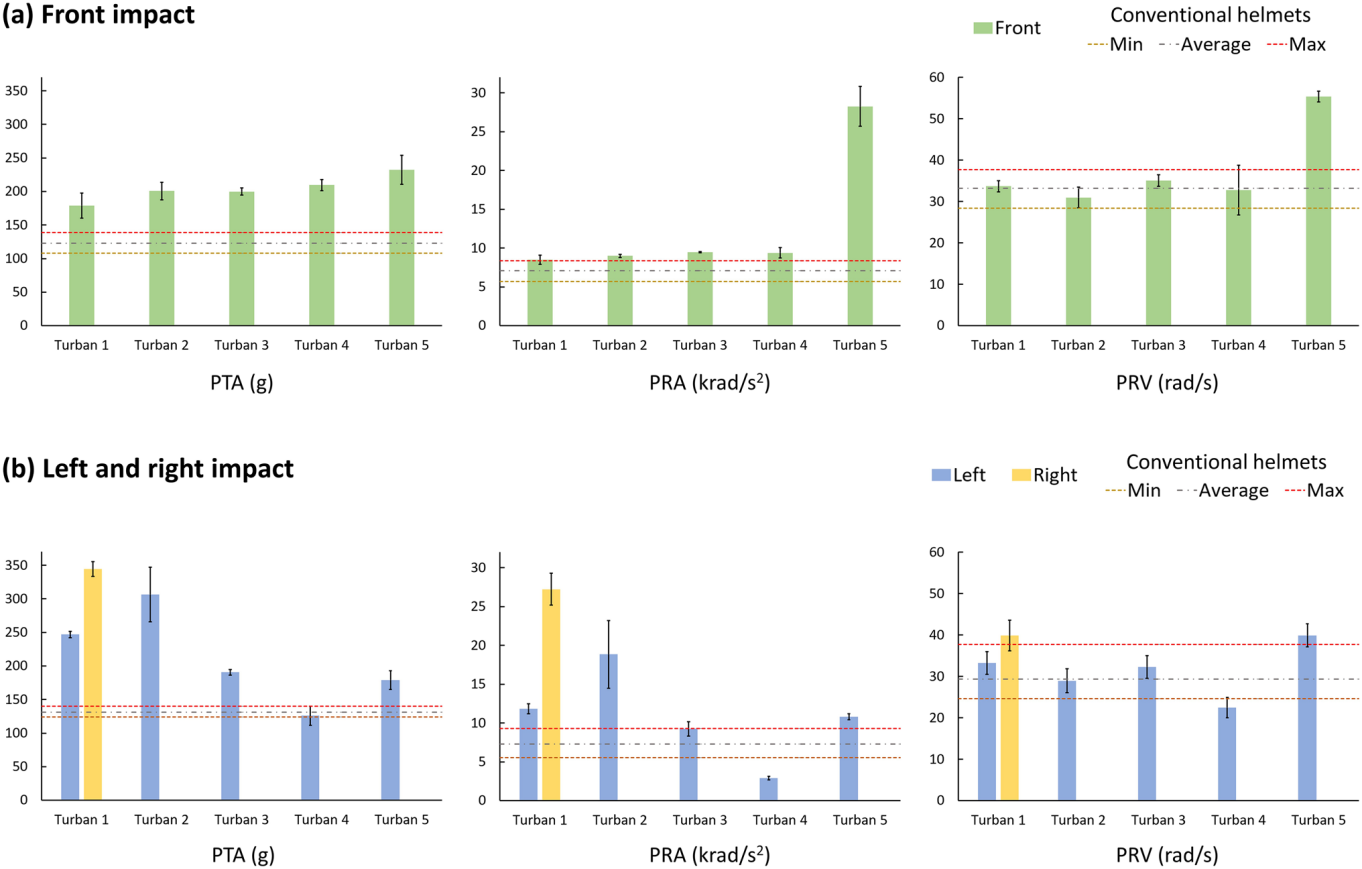
Comparisons of PTA, PRA, and PRV produced by turbans (error bars representing standard deviation) and the minimum, average, and maximum values of these injury metrics produced by the 8 different conventional helmets reported in [1]

Next, we compared the performance of turbans with bicycle helmets published in a recent study, where helmets were tested under the same oblique impacts [1]. Here, we used the results from 8 different conventional bicycle helmets, i.e., the helmets that were made of a plastic shell and an EPS liner without any other technology for mitigating head injury. Due to the symmetrical geometry of bicycle helmets about the sagittal plane, bicycle helmets have identical protective performance under left and right impacts. We used the performance of the 8 conventional bicycle helmets (i.e., the minimum, average, and maximum values of each injury metric) at left and front as baselines for evaluating turban’s performance, as shown in Fig. 5.

Under front impact, turbans produced PTA values that were 46-89% higher than the average PTA values observed with conventional helmets (Figure 5a). In contrast, the disparity in PRA and PRV between helmets and turbans (excluding turban 5) under front impact is considerably smaller. Turban 1-4 yielded PRA values that were 20–32% higher than the average PRA of helmets. Regarding PRV, turbans 1–4’s values fall within the range of values observed in helmets, with turban 2 and 4 even producing lower values than the average PRV of helmets. Turban 5 exhibited significant high values of PRA and PRV, approximately 300% and 67% higher than the average values of helmets.

During left and right impacts, turbans displayed even greater discrepancies in protective performance compared with bicycle helmets. Turbans 1–3 and 5 generated PTAs that were 37–163% higher than the average PTA of helmets, whereas turban 4 demonstrated slightly lower PTA (4%) compared to helmets. The same trend applies to PRA and PRV as well, while the other turbans produced higher or comparable values to the average value of helmets, turban 4 outperformed helmets with lower PRA and PRV values than the minimum value observed in helmets. Overall, while helmets generally offer better performance than turbans, certain turbans displayed comparable or even superior performance in one or more injury metrics. However, it should be noted that these comparisons are only valid for the impact locations tested here. Turbans may provide much less or even no protection when impacts occur at regions covered with limited amount of fabric and vice versa.

### An Analysis of the Interaction Between the Turban and Headform: Suggestions for Improving Turban Protection

Increasing the duration of impact can reduce the peak impact force, thus the peak translational acceleration of the head. Conventional bicycle helmets use foams to increase the impact duration [22]. Through the deformation of the foam, the impact duration is increased. Hence, the mitigation performance of the helmet depends on the stiffness and thickness of the foam [23]. Once the foam’s crush limit is reached, the head experiences a hard stop producing large PTA values. Similar to the helmets, turbans can increase the impact duration as the layers of fabric are compressed. However, our results show that the impact duration with the turbans is shorter than cycle helmets (10–15 ms vs 15–20 ms) [1]. This is because the fabric of the turban is easily compressed, and the layers soon bottom out during impact, thus creating large spikes in translational acceleration.

Based on the above analysis, one approach for improving turbans’ performance in mitigating PTA is to increase its crushing duration and avoiding bottoming out. There are novel bicycle helmet designs that can significantly increase the impact duration, which can guide such improvements [22]. One example is the airbag bicycle helmet called Hövding [1]. Our recent study shows that the impact duration of the Hövding airbag helmet (>40 ms) is much longer than the EPS helmets (15–20 ms), leading to a few folds reduction in PTA [1]. Another example is that the helmet liners are made of multi-layered foam with different properties [24–26]. Such designs combine high-density and low-density foams, where both foams are used to increase the impact duration, but the high-density foam absorbs more energy and avoids bottoming out in more severe impacts [22]. These examples provide possible routes for improving the performance of turbans in reducing PTA. For instance, by adding high-density foams or small airbags inside the fabric or between the fabric layers, the crushing duration can be increased and the chance of bottoming out is reduced. In addition, there are regions of the head that are only covered with a thin layer of fabric, e.g., rear and crown, which means these regions are less protected or even unprotected. Therefore, another route for improving the head protection if a turban in worn is to increase the amount of fabric in these regions.

The rotational acceleration of the headform is dependent on the interaction between the turban and headform. Head rotational motion can be produced by two mechanisms: 1. The head is subjected to non-centric impact, where the total force vector does not pass through the center of gravity of the head [27]; 2. The head is rotated by the headgear (e.g., helmet or turban) due to the fitting constrains and the friction between the head and headgear [28]. The coefficient of friction (CoF) between the turban fabric and the anvil surface are likely to be much higher than the CoF between the helmet shell and the anvil surface. A higher CoF leads to a larger tangential force applied to the turban. In addition, we observed negligible relative motion between the turban and headform in the impacts, suggesting high friction between them. These factors, in addition to the force applied to the headform, can explain the large rotational acceleration, and velocity, of the headform with turbans compared to the bicycle helmets.

For mitigating PRA and PRV, we can manipulate the factors that increase the head rotation. The majority of helmet technologies developed for damping head rotation aim on increasing the sliding motion between the head and helmet or among helmet layers [1, 7, 22]. For example, the Multi-Directional Impact Protection System (MIPS) is a slip layer between the foam and head, which allows rotational movement between the head and helmet during impact [7]. Bland et al. [29] have shown that bicycle helmets with MIPS can greatly mitigate the rotational motion, compared to non-MIPS helmets. Turbans are made of several layers of a large fabric, which create many interfaces within the turban. Adding slipping materials into the turban layers or coating the fabric to reduce friction can reduce the transmission of rotational motion to the head.

Another method for improving turbans is the layering, as indicated by our tests on the left and right sides of the same turban and tests on different turban styles. Turban 1 had different layering on the left and right sides (Figure 1a). Impacting the right side of this turban produced significantly larger PRA and PRV than impacting its left side, with 130% increase in PRA and 20% increase in PRV. This suggests that different layers at the left and right sides significantly affected the head kinematics although both sides look visually similar in size and thickness. In addition, style 2 (Dumalla) turbans produced lower PRA and PRV than style 1 (Dastaar or Pagadi) turbans under side impacts. Particularly Turban 4 (style 2) produced surprisingly low values of rotational injury metrics in left impact, which are also lower than the conventional bicycle helmets (Figure 5) [1]. These observations suggest that turbans’ protection can be improved by optimizing the layering of the fabric.

### Limitations and Summary

There are some unique characteristics of turbans under oblique impacts. First, there were no obvious damage to the turbans after each impact. Only minimal frictional marks were shown at the impact location of the fabric. This is different to bicycle helmets, whose shell and liner show obvious damage after impact. Secondly, the turbans are hand-wrapped, and their finishing are dependent on individuals. In our tests, each turban was wrapped by the same person. However, there were variations among the same type of turbans although they look similar. This may explain why the CoVs of the kinematic metric, particularly rotational metrics, across the repeats are relatively large. This is different to commercial helmets which have consistent quality and performance, leading to lower CoVs [1, 7, 8].

Our study has several limitations. First, we tested the turbans using flat anvils. This is supported by a recent literature review of real-world cyclist collision scenarios, which shows that in nearly 80% of the cases, the head impact is reported to be against a flat surface [2]. Current bicycle helmet test standards also specify impact tests onto hemispherical, kerbstone, or edge anvils, covering various real-world impact surfaces [30]. The shell of bicycle helmets can mitigate penetrations when impacting against these non-flat anvils or any sharp objects. However, turbans do not have such protective ability due to the absence of a hard shell, leading to high risk of skull fractures under impacts onto non-flat surfaces. Future work should investigate turbans’ performance in impacts onto non-flat anvils.

Secondly, we tested turbans at specific locations, which are all covered with thick layers of turbans. However, turbans do not cover the whole head with the same amount of fabric; there are regions covered with thin layer of fabric (such as the crown and back of the head). Impacts at these thin-fabric-covered locations result in head kinematics similar to unprotected impacts. To protect the accelerometers and headform, we did not test these locations. This means that the test results obtained here represent an upper bound of turbans’ protective performance. Further work should address the limitation of the sensor measurement and headform protection, allowing for more severe impact tests.

Another limitation of this study is the adoption of the HIII headform. This headform has been used in several previous studies on helmets due to its similar physical properties to the average human head [7, 19, 31–33]. However, this headform has a substantial drawback: its vinyl rubber skin has a much higher CoF against the fabric compared with the human scalp. Previous studies have experimentally determined a CoF between the human head and EPS form or polyester fabric in the 0.2 to 0.35 range, which is much lower than a 0.75 CoF between HIII headform and fabric [34, 35]. This limitation can affect the test results in two ways: 1. The high CoF between the headform and the turban fabric may lead to overestimation of head rotational measures [8, 36]; 2. The gripping between the HIII headform and turban may be higher than the human head and turban. During out tests, we observed that most turbans maintained their shape and position during the impact without coming off the head, which is probably due to the high gripping between the HIII headform and the turban. It remains unclear whether a lower level of gripping between the human head and turban will change their interaction. Besides, we used an isolated HIII headform without its neck as the Hybrid III neck has limitations. However, it has been shown that the headform‐only tests produced greater peak linear and rotational values than the tests with a neck [37]. Future work should employ headforms with a CoF that more closely mimics human head, as well as quantify the neck's effect on turbans [8, 38].

In summary, we conducted the first assessment of turbans’ protective performance under oblique impact conditions. We analyzed the interaction between turbans and headform and suggested possible ways for improving turbans for better mitigation of both translational and rotational injury metrics. The results show that turbans can reduce head injury, compared to unprotected head impact. However, such protection is limited to impact locations covered with thick layer of fabric. The results of this study can help improve the safety of a group of road users who wear mandatory headgears due to religious tenets or other reasons, such as those who might have medical or statutory reasons to cover their head instead of wearing a helmet.
